# Optimising the provision of health information for older adults across paper and screen formats – A requirement study with content producers and consumers

**DOI:** 10.1371/journal.pdig.0001090

**Published:** 2025-11-17

**Authors:** Larissa Taveira Ferraz, David Mark Frohlich, Charo Elena Hodgkins, Haiyue Yuan, Paula Costa Castro

**Affiliations:** 1 Faculty of Arts, Business and Social Sciences, University of Surrey, Guildford, England; 2 School of Psychology, University of Surrey, Guildford, England; 3 Institute of Cyber Security for Society (iCSS), School of Computing, University of Kent, Kent, England; 4 Department of Gerontology, Federal University of Sao Carlos (UFSCar), São Carlos, Brazil; University of Pittsburgh School of Medicine, UNITED STATES OF AMERICA

## Abstract

The global shift toward digital health communication presents both opportunities and challenges for older adults, whose populations is expanding rapidly. This study explored how older adults and health content producers engage with health information across paper and digital formats, and assessed the potential of hybrid approaches such as augmented paper. Two qualitative studies were conducted in Surrey, UK: focus groups with older adults (n = 9) and interviews with public health professionals (n = 6). Data were analysed through content and thematic analysis to identify user requirements. Findings show that older adults continue to value printed materials for familiarity and reliability, but turn to digital formats for timeliness and convenience. Trust in online content, ease of use, and device compatibility emerged as central concerns shaping engagement. Content producers echoed these challenges, highlighting cost constraints and the need for accessible, multi-format materials. Both stakeholder groups favoured app-free connections between print and digital content, with QR codes preferred for their simplicity, familiarity, and avoidance of app installation. Participants also emphasised the importance of multimodal presentation (e.g., text, video, audio) and options to self-print key materials. While based on a small, UK-specific sample, the study highlights design implications for inclusive health communication. Hybrid solutions that combine print with carefully curated digital resources can reduce barriers linked to trust and usability, and extend access for older adults with varied levels of digital confidence. These insights provide actionable guidance for public health organisations and policymakers seeking to balance cost-effectiveness with accessibility. Broader testing in more diverse populations is recommended to refine these strategies and ensure equitable health communication worldwide. These findings underline the importance of designing hybrid health communication strategies that are not only user-friendly but also equitable, supporting the goals of the WHO Decade of Healthy Ageing by promoting inclusive access to reliable health information for older adults worldwide.

## Author summary

As more health information moves online, older adults, who are one of the fastest-growing age groups globally, face new challenges in accessing the content they need. While printed materials are familiar and easy to use, digital formats offer benefits like up-to-date information and wider access. In this study, we explored how older adults consume and engage with health information, and how professionals working in the public health sector create and deliver it. We worked with two groups: older adults, who are the main users of this information, and health content producers responsible for its design and dissemination. Both groups interacted with a new format called augmented paper, which combines physical printed materials with digital content accessed through smartphones. Our findings show that older adults appreciate the reliability of printed materials but often rely on digital sources for convenience and timely updates. However, they face barriers such as lack of trust in online content, difficulties navigating apps, and discomfort with technology. Both older adults and professionals preferred simple, app-free solutions such as QR codes to connect paper to digital resources. They also highlighted the importance of using clear and accessible formats, like videos, images, and audio, which can cater to different learning styles. This study highlights the need for hybrid communication strategies that respect older adults’ preferences while taking advantage of digital tools. Augmented paper, when designed well, offers a promising way to improve health information delivery by combining the strengths of both formats. These insights can help make health communication more inclusive and effective for older populations with varying levels of digital confidence.

## 1. Introduction

The global population of older adults is expanding at an unprecedented rate. In 1980, there were approximately 260 million people aged 65 and over, a figure that had nearly tripled to 761 million by 2021. Projections indicate that this number will exceed 1 billion by 2030 and reach over 1.6 billion by 2050. The number of people aged 80 and older is increasing even faster than the 65 + population. By 2050, the global population of those aged 80 and above is expected to reach approximately 459 million, almost three times the 155 million recorded in 2021 [1]. This demographic transition emphasises the increasing importance of addressing the needs and challenges faced by an ageing population.

In response to the rapid demographic shift, the United Nations (UN) and the World Health Organization (WHO) have recognised the urgent need for global action. This led to the establishment of the *Decade of Healthy Ageing (2021–2030)*, a strategic initiative aimed at fostering longer and healthier lives through multisectoral collaboration [2]. Among its key objectives is the improvement of health communication, which plays a critical role in promoting healthy ageing by ensuring that older adults have access and relevant health information. By supporting health literacy and encouraging engagement with health services, clear and accessible health information equips older adults with the power to make informed choices that significantly enhance their quality of life as they age [3].

The provision of health information is increasingly shifting from printed materials to digital formats, even for older adults. While this transition offers benefits such as real-time updates and broader accessibility, it also presents challenges for older populations [4,5]. Recent studies highlight the barriers older adults face when seeking and accessing online health information, including the proliferation of low-quality content, a large volume of irrelevant information, and distracting elements such as pop-up advertisements. Additionally, many older users encounter difficulties with poor website navigability, limited accessibility features, and overly complex designs, which can hinder their ability to locate and interpret reliable health resources [5,6]. These challenges make evident the need for user-friendly and inclusive health communication solutions to ensure that older adults can effectively access and engage with health information.

A promising solution to overcoming these challenges is augmented paper, which combines the familiarity and accessibility of printed materials with the dynamic capabilities of digital media [7]. A recent scoping review highlights the shift from traditional paper to digital formats in health education interventions for older adults, while emphasising the unique advantages of both media. This suggests that an integrated approach could significantly enhance health communication for this population [4]. Printed materials continue to be preferred by many older adults due to their availability, portability, and ease of use. These materials offer a tangible and accessible format that requires no digital literacy or internet access. In contrast, digital tools provide valuable advantages, such as interactivity, multimedia explanations, real-time updates, and personalisation, which can improve engagement and comprehension [4]. Despite the challenges digital approaches may present, older adults are generally open to adopting new information and communication technologies when these tools are perceived as convenient and reliable [8]. By bridging the gap between paper and digital formats, augmented paper presents a promising, inclusive, and user-friendly solution for delivering health information tailored to older adults’ diverse needs.

This study builds on the potential of augmented paper by exploring how older adults currently engage with health information, both digitally and in print. Through focus groups and interviews with older adults and health content producers, the research uncovers key barriers and facilitators in accessing, using and producing health information effectively. By integrating these perspectives, the study brings insights into the development of user-centred health communication solutions that combine both digital and print media.

The aim of this research is to understand the user requirements for effective health communication solutions between paper and digital formats for older adults. This includes exploring current practices in both the consumption and production of health information, as well as understanding the potential role of augmented paper in this context. The study addresses three key questions: 1) How do older adults currently consume health information through paper and digital formats? 2) How is health information produced in these formats? 3) What role can augmented paper play in health communication for older adults? By answering these questions, the research provides valuable insights into how health communication can be made more inclusive, accessible, and user-friendly, offering practical recommendations for future solutions.

## 2. Materials and methods

This research incorporates two qualitative requirement studies involving key stakeholders: a focus group study and an interview-based study to investigate health education for older adults. The focus group study aimed to explore older adults’ experiences and attitudes toward health information, providing insight into their needs and preferences. In parallel the interview-based study gathered perspectives from professional content producers working in public health to understand the strategies and challenges involved in creating health content for this audience. This dual-stakeholder approach allowed for a holistic exploration of both the consumption and production of health information, offering a more complete understanding of health communication dynamics for older adults.

This study received a Favourable Ethical Opinion by the University of Surrey Ethics Committee (Ref: FASS 22–23 096 EGA). Both methods in this article involved a presentation of an interactive document (prototype). In the following section, we explain its features and describe how it was used in each method.

### 2.1 Prototype development

A prototype was developed to demonstrate how interactive health education can be delivered by integrating traditional paper and screen media, referred to as ‘augmented paper’. This involved using the *‘Next Generation Paper’* (NGP) platform, developed at the University of Surrey, to link digital content on a smartphone with printed hotlinks on a document [9].

The augmented paper used in the demonstration was created using the *Authoring app* and experienced through the Player app. Both applications were developed as part of the NGP project by the University of Surrey to support the creation of augmented paper.

The NGP platform was selected for this study because it allowed a seamless link between printed materials and multiple forms of digital content (text, video, audio, web links) without requiring specialist equipment. Alternative augmented print technologies, such as marker-based augmented reality (AR) or app-dependent interactive print platforms, were considered but were less suited to our study aims. These often require the installation of proprietary applications, higher levels of digital literacy, or additional hardware, which could have introduced further barriers for older adult participants. By contrast, the NGP platform offered a lightweight, user-friendly solution aligned with our goal of exploring low-threshold, inclusive forms of hybrid health communication.

**Fig 1:**
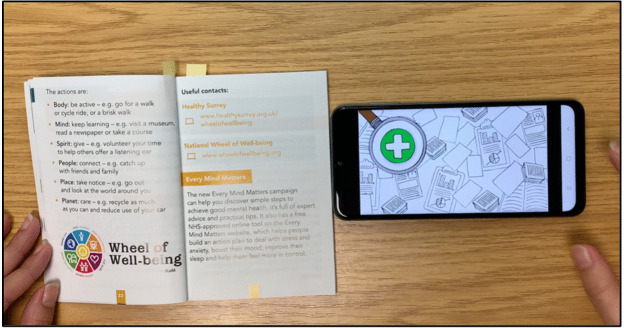
Augmented paper prototype.

The prototype was developed by augmenting “*First Steps: Emotional Health and Mental Well-being”*, a public health booklet created by Surrey County Council (SCC) in partnership with the National Health Service (NHS). This booklet was chosen as it provides preventive health information and incorporates multiple web links. Using the NGP platform, printed pages were linked to multimedia resources (videos, audio, images, web content), which could be accessed via a companion mobile app (Fig 1). Detailed navigation functions and technical specifications are provided in S1 Appendix.

**Fig 2:**
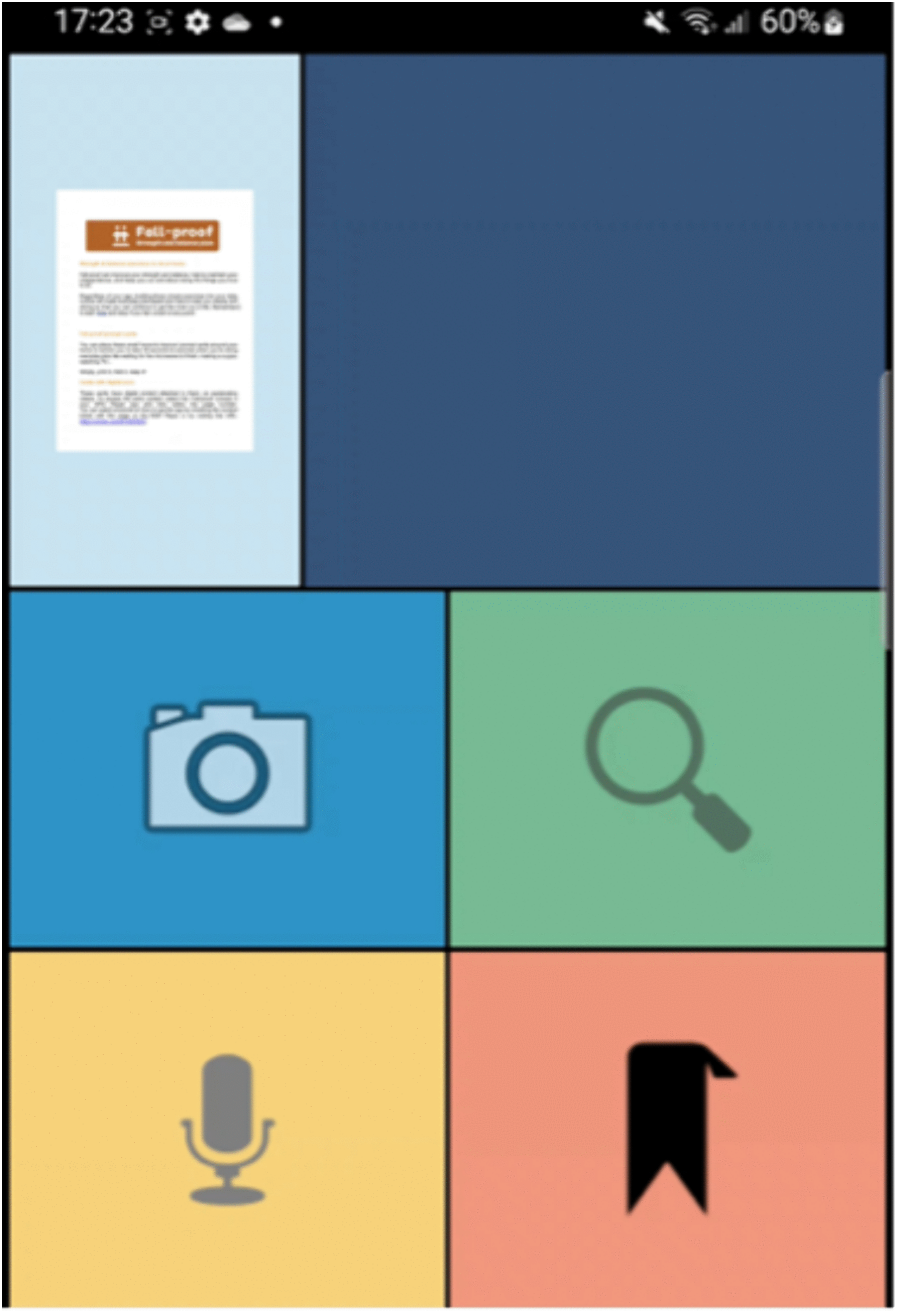
The home and pages interface.

**Fig 3:**
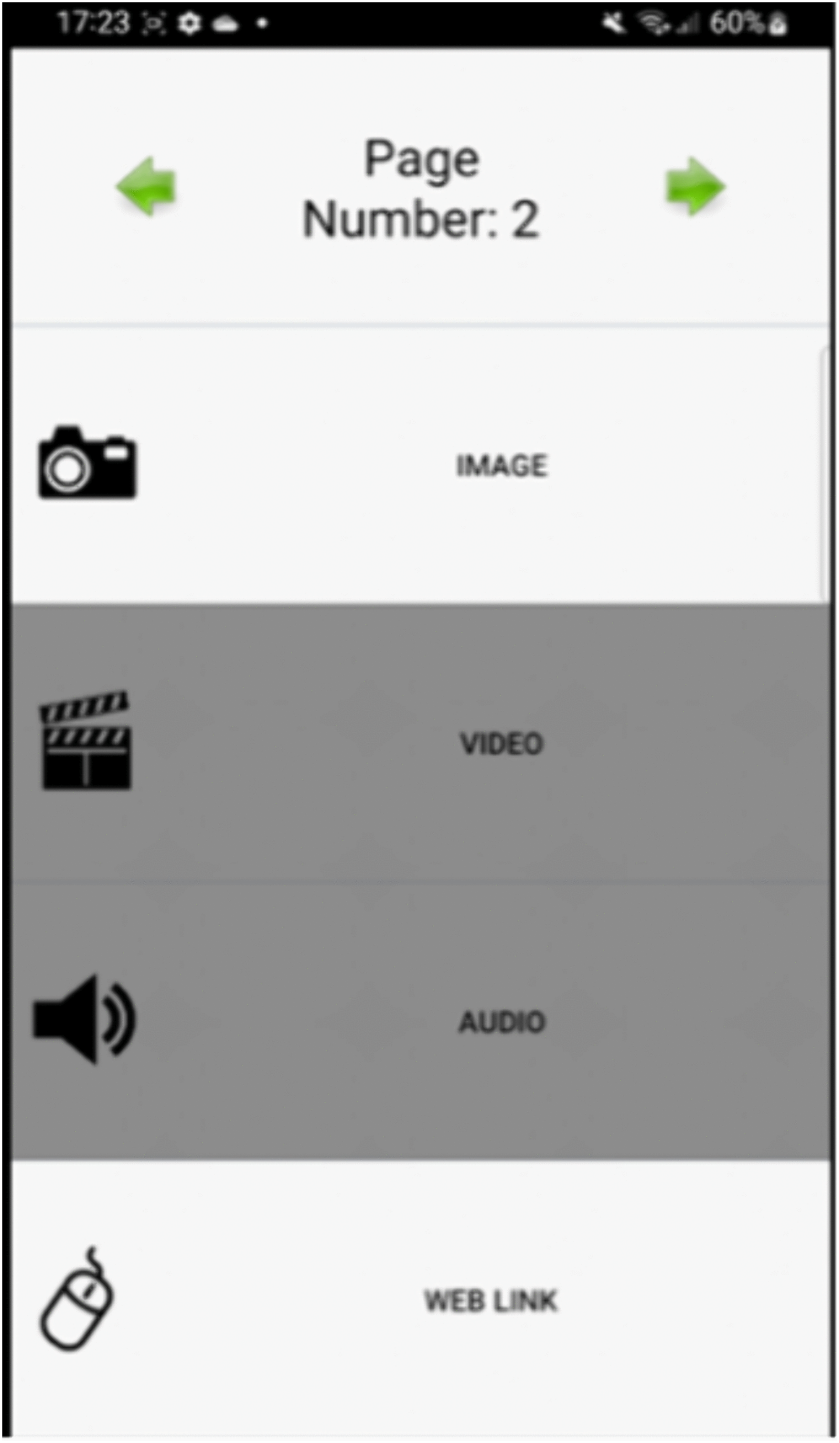
Interface of the augmented paper Player app.

The prototype demonstration included a series of interactive health information pages featuring diverse media content, such as audio recordings in multiple languages embedded on individual pages, educational videos, hyperlinks to NHS and SCC resources, images, infographics and a video guiding a breathing exercise. For example, when using the paper-screen link, a person can click the microphone button and say the number of a page (Fig 2). They are then given the option to either listen to an audio reading of the page’s content or view an image that provides a visual explanation of the information presented in the written text (Fig 3). All content used as hotlinks were sourced from the SCC website. In the next two sections, we provide further details on the study methods and how the prototype was used in each.

### 2.2 Focus group

The focus group study involved in total nine older adults aged 60 years or older, all residing in Surrey, UK. Although twelve participants were originally recruited, three were unable to attend on the day due to personal reasons. To accommodate availability and ensure sufficient speaking time for each participant, two comparable mini-focus groups were conducted by Author 1 and 2: one with four members and another with five. Sample size was determined with reference to established qualitative research guidance, which indicates that small, homogeneous groups are sufficient to achieve thematic saturation in focused requirement studies of older adults [10].

Participants were recruited through the *Surrey Healthy Ageing Research Partnership* network. Inclusion criteria required participants to be aged 60 years or above, fluent in English, own a smartphone or tablet, and be able to attend an in-person session at the University of Surrey. They were also asked to self-report sufficient cognitive and functional ability to engage in group discussion. No minimum prior level of digital experience was required, although participants generally reported moderate familiarity with using digital devices. Demographic information collected included age, gender, and education level; however, socioeconomic and ethnic diversity were not systematically captured, which we acknowledge as a limitation. Each participant received a small incentive in recognition of their contribution.

A semi-structured discussion guide was developed to facilitate the session. The discussion guide was divided into two parts. The first part contained questions related to their current forms to access or receive health information. The second part included questions related to their impressions and feedback about the demonstration on the augmented paper prototype.

The sessions were conducted face-to-face in a designated room at the University. After a brief introduction explaining the study’s goals and time for participants to sign the consent form, the first part of the discussion was initiated with the questions presented in Table 1:

**Table 1:** Discussion guide: Current forms of accessing or receiving health information.

Question No.	Question
1	How do you currently access health information?
2	What are the paper formats in which you access health information?
3	What are the screen-based formats in which you access health information?
4	What are your preferences regarding these formats?
5	What are the barriers you experience when using these formats?
6	Do you keep the content for future consultation? If so, how?

Following the first part of the discussion, participants took a 10-minute break, before beginning the second part of the session. The augmented booklet prototype was then introduced through a short scripted demonstration by the researchers, after which participants were given time to explore it individually. The researchers adopted a facilitative style, guiding the discussion with pre-defined, semi structure interview (Table 2), while avoiding leading prompts, in order to minimise researcher influence on participants’ responses. The facilitation approach emphasised clarification and prompting for depth, rather than directing participants toward particular viewpoints. Data were collected through both audio and video recordings to ensure accuracy of transcription and to capture non-verbal interactions with the prototype.

**Table 2:** Discussion guide: Impressions and feedback on the augmented paper prototype.

Question No.	Question
1	What were your impressions about the demonstration?
2	What was the best aspect? Why?
3	What was the worst aspect? Why?
4	How usable do you think this link between paper and screen could be?
5	How could it be improved?
6	What other device might be used to display content?

Each part of the session lasted approximately 50 minutes, and the whole session lasted approximately 2 hours. The sessions were audio and video recorded, with participants’ consent, to ensure accurate transcription.

### 2.3 Interview study

The interview-based study included six professionals involved in the content production for Surrey County Council (SCC) - Public Health. All participant roles were involved in creating or managing preventive health content. Participants were purposively sampled to reflect a range of professional roles, including programme leads, communication managers, and content designers. This ensured that strategic, operational, and design perspectives were all represented. Recruitment was organised in partnership with SCC and participants were invited to take part in an online interview via Microsoft Teams, lasting approximately one hour.

Sample size was determined with reference to qualitative research standards, which suggest that small, purposive samples can be sufficient to capture the perspectives of distinct professional groups and to achieve thematic saturation [11]. Saturation was assessed during analysis and judged to have been reached by the sixth interview.

The interviews captured a comprehensive range of thematic insights relevant to the research questions (RQ), consistent with established criteria for achieving code saturation in applied qualitative research.

A semi-structured interview guide covered two areas: (1) participants’ professional practices in producing and disseminating health information, and (2) their impressions of the augmented paper prototype. All the interview sessions were conducted by Author 1.

Each interview began with a brief overview of the study goals, and then the first part of the interview was initiated with the questions presented in Table 3:

**Table 3:** Interview Schedule: Creation and distribution of health information.

Question No.	Interview Question
1	What is your role in the creation of health information?
2	What are the phases of the process?
3	What leads the choice of paper and screen channel distribution?
4	What are the challenges in the creation of content and distribution in each media of health information?
5	What are the challenges and opportunities to combine paper and screen?

Following the first part of the interview, the prototype was introduced (S1 Appendix) via a short scripted demonstration using a video demo, and then participants engaged in the second part of the semi-structured interview (Table 4), focusing on their impressions of the prototype. The interviewer adopted a facilitative style, asking clarifying questions while avoiding leading prompts. This approach sought to minimise researcher bias and encourage participants to share their perspectives freely.

**Table 4:** Interview Schedule – Impressions and feedback on the augmented paper prototype.

Question No.	Interview Question
1	What were your impressions about the demonstration?
2	What was the best aspect? Why?
3	What was the worst aspect? Why?
4	How usable do you think this link between paper and screen could be?
5	How could it be improved?
6	What other device might be used to display content?

The interviews lasted approximately 60 minutes and Microsoft Teams calls were digitally recorded with the participants’ consent.

### 2.4 Analysis approach

Data were analysed using a two-stage qualitative approach. First, a inductive content analysis [12] was conducted independently for each study to systematically code and categorise participants’ responses. This inductive process involved repeated readings of transcripts and notes to ensure familiarity with the data, followed by manual coding and grouping of similar content. NVivo software supported data management and organisation. In the second stage, a more deductive thematic analysis [13] was undertaken across both studies, aligning emergent codes with the research questions to identify overarching themes and cross-cutting issues.

Initial coding and cross-checking were conducted by Author 1. The grouping of codes into themes was collaboratively reviewed and discussed by Authors 1 and 2. Final thematic interpretations and conclusions were then collectively discussed and refined through critical dialogue among Authors 1, 2, 3, 4 and 5. Discrepancies were resolved through comparison and discussion until consensus was reached, supported by regular team meetings to ensure consistency. Formal assessment of thematic saturation indicated that no new codes emerged during the second focus group, suggesting that saturation had already been achieved. Subsequent interviews confirmed this, with no additional themes arising, and recruitment ceased accordingly. This dual-stage analytic process enhanced both the credibility and transparency of the findings.

Ethical approval was granted by the University of Surrey Ethics Committee (FHMS 22–23 029 EGA). Confidentiality was preserved through pseudonymisation of transcripts, removal of identifiable details, and secure encrypted storage of data on university servers, with retention in line with institutional policy.

## 3. Results

The results of this dual-method requirements study are presented in three sections: findings from the focus group study, findings from the interview-based study, and an integrated analysis of the results, highlighting the user requirements identified in both studies.

### 3.1 Focus group study

#### 3.1.1 Participants’ demographics.

Nine older adults aged 60 and above participated in the focus group study, including five women and four men. All participants were English-speaking residents of Surrey, UK, with ages ranging from 64 to 87 years. Details of their demographic characteristics are presented below in Table 5.

**Table 5:** Participants’ demographic characteristics.

		Participants
Gender	Female Male	5 4
Age (years)	Mean Median Min Max	70.55 68 64 87
Education level	Post Primary Education Undergraduate degree Postgraduate degree	2 4 3

#### 3.1.2 Results from the questions about current forms of accessing or receiving health information (Table 1).

**Seeking and receiving health information:**

All older adult participants in the focus groups reported that they primarily seek health information by searching on Google. A few also mentioned consulting their spouse about health-related questions. Regarding online search, the NHS website was frequently referenced as a reliable and trustworthy source of health information. Additionally, some participants mentioned using Google Lens to search for information about visible health concerns, such as rashes or moles.

When it comes to actively seeking health information, participants stated that they typically search for information only when experiencing symptoms or illness

They indicated that a medical doctor would be consulted only if the issue seemed more serious or if symptoms persisted. Notably, none of the participants mentioned seeking preventive health information in the absence of specific health concerns.

When receiving health information, participants said that they often consume such content through various media sources, including TV health programmes and news websites like the BBC. Much of this information relates to healthy lifestyles. Participants also noted that their GP surgeries contact them via text message or mail to invite them for vaccinations or health screenings. One participant mentioned receiving targeted health education related to their diabetes diagnosis. This included printed materials and a webinar on maintaining a healthy diet, both provided by their GP.

**Trust:**

Trust emerged as a significant concern during the group discussion. Many participants emphasised the importance of relying on official websites, such as the NHS website, or reputable sources like charities dedicated to specific health issues when searching for health information. They highlighted that “trust is a big thing,” particularly given the increasing prevalence of scams. One participant recounted a situation where they received a message from the NHS containing a weblink to check their eye test but hesitated to click on it, fearing it might be fraudulent. This concern was captured in their statement: *“In some documents, they’ve got links in there, it’s about how do you trust the source of that link? Yes. And that’s a hard thing, and I think that’s where you get some information come through from the hospital, the other day... They diverted me to a link for my eye test, and I was a bit worried about whether that was a scam. Because you don’t… They didn’t say that there was just this link that you’d go to your report. And I think if I hit that and then went the wrong way… So trust is a big thing. I think these days, and even more so because scams are getting more frequent, yeah.”*

The spread of false and misleading health information on social media was mentioned as a significant concern. One participant shared an example of a family member with a health condition who frequently consumes information from unreliable online sources. Additionally, participants expressed scepticism about fully trusting online health systems, citing concerns that these systems can sometimes fail or lose important information. This lack of trust highlights the limitations of relying solely on digital platforms for health-related content.

**Sources and formats of health information:**

Participants noted that accessing health information in paper format is less common in their daily lives than in the past, but it remains present in specific situations. They reported receiving printed materials from healthcare professionals after medical examinations, such as information about managing diabetes, or before undergoing procedures, such as leaflets explaining the process of an operation. Additionally, they mentioned receiving travel-related health information from travel nurses prior to trips abroad. Participants also observed the availability of leaflets in doctors’ clinic waiting rooms and pharmacies. Furthermore, they mentioned receiving letters at home from the NHS, including health appointment confirmations and invitations for screenings.

With regard to digital formats, smartphones were described as the most convenient option for quick health searches, particularly when prompted by something participants saw or heard, such as a programme or conversation. Participants also reported consuming health information through news articles on their smartphones. While some mentioned using desktop and laptop computers, these devices were preferred for conducting more thorough and specific searches. Other methods of accessing health information included listening to the radio while in the car, watching health-related television programmes, and listening to health-focused podcasts. A few participants owned tablets, but these were primarily used for reading e-books and not for consuming or receiving health-related content.

**Preferences about media formats.**

Smartphones were highlighted as a convenient tool for accessing health information, as participants noted they are always readily available in their pockets. One participant explained: *“Yeah, I would use my phone more than my laptop. Just for the convenience that my phone is always in my pocket, whereas my laptop I would have to go to my….’* Another added: *“It’s kind of ohh... I wonder… and you know the phone comes out and you just quickly… You know, something might stimulate my interest on a TV programme I’m watching or something. And I think, oh, well, I need to learn a little bit more about that.”*

Participants expressed a preference for receiving information from the NHS electronically, citing instances where receiving physical letters was inconvenient, particularly when they were travelling and unable to access the correspondence. Some participants also favoured digital formats for environmental reasons, noting that it reduces paper waste. Furthermore, they emphasised that online devices are the most effective option for conducting quick searches and accessing up-to-date information. One participant mentioned that while a smartphone’s smaller screen is suitable for reading brief information, the larger screen of a laptop is preferred for more extensive content, as it displays more information at once.

Some participants expressed a preference for paper when engaging in careful reading, as they found printed materials better suited for reading slowly, reflecting on the content, and revisiting it later. Paper was also valued as a reliable format for reference materials and personal records, offering a sense of physical accessibility. Participants appreciated the convenience of having documents in a specific, easily accessible location within their homes. One participant explained: *“The fact that I can pick it up and put it down quickly, and I know exactly where it is, such as an insurance document… you know… The booklet… if I have to make a reference to it, I don’t have to get on to the website, find the right place, go log into passwords. I’ll pick up that book, and that’s where it is. I know… Information that I need specifically that I know is in to-hand paper.”*

**Comparing paper and digital information retrieval**

A key topic that emerged during the group discussion was the contrast between accessing information on a screen and the simplicity of retrieving it from paper. Some participants expressed frustration with the digital experience, describing it as time-consuming to relocate information they had previously seen online. They also highlighted challenges such as forgetting passwords, which often discouraged them from continuing their search for desired information. Conversely, a few participants noted that they found it more challenging to remember where they had stored printed materials at home, suggesting that conducting another online search might be easier for them in such cases.

One participant highlighted the contrasting advantages and drawbacks of paper versus digital formats, explaining: *“I think there’s a certain amount of information... It’s about the ability to retrieve that information, and if you’ve got something printed out and filed away somewhere, you know that you can put your hand on it. But at the same time, you risk it not being up to date. So, I think generally… If it’s reference material, I’d prefer paper to be able to refer to. However, I would also, for the sake of getting the most up-to-date information, go online to get that information. But it would frustrate me if I had to, as you were describing, you know, to set up the computer, go through the passwords, try and find the document you were looking for and things like that. Whereas, you know, you can go straight into a room, open a cupboard... and you know where it was.”*

**Challenges of digital and printed health information**

Participants highlighted several drawbacks of relying solely on digital formats for health reference materials. They noted that finding the same information again can be time-consuming and lacks the tangibility of paper. Additionally, the participants expressed concerns about the potential failure of digital devices, such as computers malfunctioning, smartphones freezing, or information being lost, which reinforces the continued relevance of paper. They also acknowledged socioeconomic disparities, recognising that access to smartphones, tablets, or computers varies based on financial means. Furthermore, some participants raised concerns that excessive reliance on digital interactions could become a social barrier, reducing opportunities for personal engagement.

A shared experience among the group was the disappointment of receiving emails or encountering advertisements about health topics that ultimately turned out to be sales pitches for remedies or vitamins. This frustration prompted many to exercise caution when engaging with such content. They noted efforts to avoid suspicious links and decline cookie permissions to minimise the likelihood of receiving similar promotional material in the future.

The challenge of logging in and remembering passwords was frequently mentioned as a barrier. One participant illustrated this frustration, stating:

*“The problem I encounter very frequently, probably twice today, is passwords. Whatever happens, it’s always, ‘Yeah, please give your password.’ I haven’t got a clue. I’ve got a notebook full of passwords, which I’ve collected over the years. I mean, I know some, but there are many that I don’t. And I find that off-putting. I just don’t consume them.”*

Technical compatibility issues were also highlighted, with one participant recounting difficulties accessing specific attachments sent from an Apple device to a Microsoft device. In addition to compatibility concerns, participants mentioned challenges with small keyboards and the closeness of the keypads, which can make typing cumbersome. One participant shared their experience with typing:

*“Yeah, I’m a one-finger person. When I type a letter, which is pathetic when I see the family, they’re whizzing away, not only just on a computer but on a little mobile. I think the majority of you are, I guess you are, but I could not possibly do that. So this inhibits me from going on to putting things on the Internet. I have to rely upon this because I’m so painfully slow, and older people, I think, fall into that category.”*

Participants also highlighted that a key challenge with paper formats is the potential for clutter and the need for significant storage space to accommodate an accumulation of printed information over time. One participant described this challenge, stating: *“There are battles at home between my wife and myself because she would like it to be nice and tidy, and I want to have all my folders, and I do not want them to be sold. Goodness knows how many years I go back, 20 years, perhaps, for various things. Because I’m… alright. I’ll say I don’t throw things away.”*

**Ways to store personal health information**

When it comes to storing health information, most participants mentioned maintaining a physical filing system for health records they consider important. These records include exam results, surgery details, vaccination certificates, prescriptions, hospital visit summaries, diagnostic letters, and medication schedules. Participants highlighted the value of keeping these physical files for easy reference, particularly to recall specific dates or access essential information such as their NHS ID number.

Some participants expressed a preference for maintaining physical health records due to their tangibility, viewing them as a reliable history of health events. They found physical files easier to organise and locate compared to digital records. Additionally, having physical copies provided a sense of security, particularly in case of issues with digital files in the NHS system. This reassurance was reflected in one participant’s statement: *“I print off everything. As I mentioned earlier. So I can go back and track down what is happening, and I found that very reassuring. I have multitudes of folders like this, and I go to those and thumb through in order to search out.”*

Opinions about storing health information on digital devices varied among participants. One participant expressed trust in the NHS storage system, stating that saving exam results was unnecessary because they could access them via the NHS app. Other participants acknowledged knowing how to organise documents on a computer but admitted that their digital folders often become cluttered, with personal files grouped together, making it harder to locate specific documents later. Some participants also mentioned that while they save information digitally, it tends to be related to leisure activities, such as holiday planning, rather than health-related information.

One participant shared their experience of transitioning from keeping physical records to using digital systems: *“I used to keep so much paper when I was at work. But I don’t need to now. You know, I can quite, quite easy… I keep a lot on OneNote. You can scan in, send photos, send documents, send links to it so that I can just file. I’ve got all my files on my holidays levels, and I saved the link so I can just go into the link and then find out where the original information came from. So I do tend to do a lot of it.”* This participant also noted that their digital filing system includes doctors’ notes and records of their health condition, which are synced with their smartphone and stored in the cloud for easy access and organisation.

#### 3.1. 3 – Results from the Impressions and Feedback on the Augmented Paper Prototype Questions (Table 2).

**Feedback - Impressions about prototype demonstration**

After the prototype demonstration, participants shared their initial impressions, noting that the app did not feel intuitive or visually appealing. They found the navigation somewhat time-consuming and expressed uncertainty about how to use the interface effectively. When accessing weblinks through the app, participants encountered warnings that the content was not secure, which discouraged them from engaging with it. Regarding the bookmark feature, participants viewed it as an unpopular idea, suggesting that users might struggle to understand its purpose.

However, participants also expressed positive impressions of the demonstration, particularly appreciating the concept of augmented health information. They found the idea of having additional, easily accessible digital information with paper valuable and liked the connection between physical and digital mediums. They highlighted that this approach could cater to diverse learning preferences, making health content more accessible to a wider audience. Participants also appreciated that digital content, within the combined paper-and-screen concept, could be updated and displayed on larger screens, not limited to smartphones. They noted that augmented technology could offer varying levels of depth, linking users to relevant information and potentially enhancing interaction and engagement with health content.

**Best prototype aspects**

Participants noted that the videos embedded in the augmented booklet appeared highly professional, with good sound quality and an appealing visual style. They appreciated the video display size, describing it as appropriate. They also highlighted the value of presenting information through diverse mediums, recognising that people learn and absorb information in different ways. While written content may work for some, others may benefit more from visual formats. For practical activities, such as breathing exercises or physiotherapy routines, participants emphasised the importance of instructional videos, as they can help users recall and perform the exercises correctly.

The group discussion also touched on the depth and increased level of interaction offered by augmented content. One participant described their experience with the concept, stating: *“I really was involved because I think that… for me, the augmented bit is about trying to increase your interaction. Yeah, and stimulate your broader sense of awareness, including giving you pictures, giving you diagrams, giving you activity. And that, I think, is… it brings to the party, as it were.”*

**Worst prototype aspects**

Navigating the Authoring app was described as unintuitive and somewhat cumbersome, with users noting it would take time to learn. The interface was considered confusing, as the oversized boxes on the first page gave the impression of being more important than the subsequent pages where content was listed for selection. Some felt that the augmented content concept was unsuitable for larger booklets, like the one used in the prototype. Instead, they suggested that augmented content would work better for smaller printed materials, such as leaflets, while larger booklets should remain complete and self-contained.

**Usability of the link between paper and screen**

The link between paper and screen was seen as having significant potential and a wide range of applications. It was suggested that this technology could be used for educational purposes by incorporating questions after content explanations to assess comprehension. Additionally, participants proposed that such interactive content could be displayed in GP waiting rooms for patients to engage with while waiting. On a broader scale, they highlighted its suitability for organisational use, such as preparing employees for retirement. For example, it could enhance retirement workshops or pension programmes by linking to detailed information about pensions and associated benefits.

**Suggestions on how to improve the prototype**

A key suggestion for improving the prototype was integrating QR code technology to streamline the connection between printed materials and digital devices. Participants unanimously agreed they would appreciate the convenience of accessing content simply by scanning a QR code. However, concerns were raised about the safety of using QR codes. Some had heard of scams involving fake leaflets with malicious QR codes designed to steal personal information or situations where fraudulent QR codes were placed over legitimate ones on official materials.

Other suggestions included incorporating quizzes after educational content to help reinforce understanding and retention. Participants expressed interest in augmenting all NHS leaflets, particularly those with practical applications, such as physiotherapy exercises, as this could enhance user interaction. They emphasised the importance of ensuring that all content is visually appealing, easy to read, and user-friendly for a comfortable experience.

**Other devices to display augmented health content**

When considering alternative devices for displaying augmented health content, participants suggested screen casting the content onto a television, which they felt could be particularly useful for group discussions involving family members or carers. Some participants also expressed a preference for accessing augmented content on a laptop or desktop, as they find larger screens more suitable for longer reading sessions.

**Print to remember**

When discussing the use of augmented paper technology for health information, participants noted that they do not necessarily feel the need to receive everything in printed form. Many expressed a preference for receiving information electronically, allowing them to decide what to print and retain as a hard copy. This reflects a balance between reducing paper waste and acknowledging their preference for printing as a way to ensure important information is readily available and easier to remember.

**Searching health information for others**

The role of participants as carers was discussed, highlighting situations where they search for health information on behalf of friends or family members and then share it with them. Depending on the circumstances, they may forward the information digitally or print it and deliver it in person. Participants appreciated the potential of using augmented paper in this context, as it allows them to provide a printed document with essential information while offering the option for the recipient to explore more in-depth digital content if desired.

### 3.2 Interview study

#### 3.2.1 Participants’ demographics.

The study involved six participants who held diverse positions within public health and communication sectors. Their roles ranged from leadership positions, such as Public Health Leads and Programme Leads overseeing mental health, health protection, and nutritional strategy initiatives, to specialised communication-focused roles, including Communications Account Managers and Content Managers. Collectively, these roles demonstrated a balance of strategic oversight and public engagement, providing valuable insights into addressing complex public health challenges.

#### 3.2.2 Results from the Creation and Distribution of Health Information Interview Schedule (Table 4).

**Phases of health information creation and distribution**

The participants described the phases involved in the creation and distribution of content within SCC Public Health. While specific processes varied slightly across departments, all followed a similar structure: identifying needs, drafting content, reviewing and approving the material, and finally distributing it through digital or printed formats.

In the phase of identifying information needs, these are derived from various sources, including the national campaigns and awareness calendar, documents containing new evidence or government guidance, and internal briefing materials that provide background information and data on prevalence in specific areas of Surrey. During the content drafting phase, teams consider insights about the target audience, including their needs, the problems to address, and the most effective channels to reach them. Once the content is created, it is reviewed to ensure alignment with national guidelines before being published online or printed for distribution.

They also collaborate with charities and their target audience to ensure that messaging is relevant and resonates with those it aims to support. One participant described this process: *“For example, I’ve been working on some promotional material around a programme that helps people with multiple disadvantages, such as homelessness, substance use, and mental health challenges. We worked with them to develop and design messaging to ensure it reflects the audience. Similarly, we recently collaborated with a charity for a gambling campaign. We attended one of their groups, discussed the messaging we intended to use, and developed it with them to ensure it was relevant and resonated with their experiences”’*

**What leads the choice of paper and screen channel distribution**

The interview participants, who work in the stakeholder company, emphasised that their insights into the target audience are crucial in guiding the choice of distribution channels for health information. They utilise data, statistics, and reports from databases such as Surrey-i to inform their strategies. Additionally, participants noted that SCC is developing a research team to enhance understanding of resident personas within the Surrey area. They acknowledged challenges such as addressing pockets of social deprivation in Surrey and effectively reaching older audiences, particularly those aged 80 and above. The participants’ awareness of these challenges is reflected in the following quote: *“Quite often in health, we are very aware of who that audience is, using health information about where people are and what the issues are. So we look at audiences geographically as well as demographically, and we target them in lots of different ways.*”

The stakeholders tailor the content and distribution channels to the intended audience, ensuring health information is accessible to all. For digital health information, they conduct user research to identify the most effective media platforms for reaching their target demographic. Campaign strategies are adapted accordingly, such as advertising on websites frequently visited by the intended audience. For younger audiences, they prioritise social media platforms like Snapchat, Instagram, and YouTube. Additionally, all content produced is made available on the Surrey Health website.

Participants noted that the use of printed health information has decreased over the years, also considering high distribution costs. However, they acknowledged that a segment of the population, particularly those who don’t access the internet, may benefit more from printed materials. This need is highlighted in the following quote: *“A few years back, we conducted a resident insight study to determine whether we still need to produce a paper version, and the response was a strong yes. It is still heavily used; we know this because we print thousands of copies every year, distribute them all, and then need to reprint another batch. The beauty of something like that booklet is that it’s portable, it can fit in someone’s pocket.”*

Regarding printed materials, participants noted that while printing itself is relatively inexpensive, the cost of mailing these materials to residents’ homes is a significant expense for the Council. Depending on the target audience and the campaign, information is distributed in both digital and printed formats. All content is accessible on the website and can also be printed as needed. For high-priority campaigns, such as vaccinations, advertisements are shared online, while leaflets are made available in libraries, community centres, and family centres. Additionally, participants mentioned that they still receive requests for paper versions of health information, and some staff use printed materials, such as booklets, to distribute directly to individuals accessing their services. The choice of format ultimately depends on the urgency and importance of disseminating the information.”

**Challenges on the creation of content and distribution in each media on health information**

Participants highlighted challenges in creating and distributing content, noting that it is not just about disseminating information but truly engaging the audience. They explained that merely presenting the facts about health topics can sometimes come across as ‘preachy’ and focus too heavily on sacrificing, which may not be effective. Instead, they emphasised the importance of making people feel they are gaining benefits or fostering a sense of community. Initiatives like group smoking cessation programmes, walking groups, cooking healthy meals together, and sharing new recipes are examples of activities introduced by SCC that can often be more impactful than health information alone.

The interviewees emphasised the importance of ensuring that health information is grounded in robust and up-to-date scientific evidence, while also catering to the diverse needs of different audiences. Participants recognised the challenge of tailoring messages to various groups, acknowledging that people receive and interpret information differently. This awareness is reflected in the following quote: *“So they don’t receive all those messages in the same way. What are you saying to people with learning disabilities? And what are you saying to people who can’t afford to go and join the gym or who are facing a cost-of-living crisis, struggling to pay the mortgage or the rent? You definitely have to bear in mind your audience, and I think, yeah, we try and do that.”*

One challenge raised regarding digital information and advertisements is their fleeting nature. Although the number of accesses in digital campaigns can be measured, there remains uncertainty about whether people genuinely notice the message or simply scroll past it. This challenge is illustrated in the following quote: *“With digital… you know, it can take quite a while for people to click or engage with a digital ad, and it depends on how people are consuming. You know, when they’re on their phones, they might be on the train or whatever, and they’re kind of like, oh, I’ll look at that later.”*

The use of physical hard copies for health information has been declining as stakeholders have become more selective about distributing printed materials. However, this format remains valuable for reaching audiences who may not engage with digital channels. A key challenge with printed content is ensuring it stays up to date, users may encounter outdated leaflets with incorrect contact details or information about services that have since changed. In contrast, digital channels offer the advantage of being easier to update and avoid wasting physical resources.

Adhering to the specific guidelines for different media formats is an important consideration in the creation and distribution of health information. This distinction is highlighted in the following quote: *“There’s a difference between web copy and print copy in terms of how the links are displayed on print, so… That in mind, it wouldn’t be the same text for both. And again, like there’s the accessibility guidelines for web copy as well and offline. Those guidelines would be slightly different. So there are various considerations and differences with both.”*

**The combination of paper and screen and its challenges**

SCC employs a combined approach to distributing health information through both paper and digital channels. All health content is made available online, allowing individuals to access and print it; however, it is important to note that this option is only feasible if they have access to a functional home printer. In addition, some materials produced by SCC are printed by them and distributed to health partners, who then share the information with patients. However, these printed materials are also accessible online, enabling the public to choose their preferred method of accessing the information based on their needs and circumstances.

An approach mentioned in the interviews that bridges paper and digital channels for health information is the use of QR codes, which cater to diverse audience preferences. While some individuals may favour printed materials, others may prefer accessing additional digital content via their smartphones. QR codes provide the flexibility to choose between these options. However, it is important to note that accessing the digital content requires a smartphone and internet connection, which may be a limitation for some. In such cases, the printed materials ensure that essential health information is still accessible. Additionally, QR codes enable stakeholders to track the number of accesses to digital campaigns, offering valuable insights into audience engagement.

#### 3.2.3 Results from Impressions and Feedback on the Augmented Paper Prototype Interview Schedule (Table 4).

**Feedback - Impressions about prototype demonstration**

After viewing the video demonstration of the prototype during the interview sessions, participants shared positive initial impressions. They described the prototype as engaging and interactive, appreciating how digital media offers alternative ways to present information beyond traditional reading. They valued the printed content as a reliable reference while recognising the benefit of providing access to additional content through digital channels. Participants noted that increased engagement with diverse formats, such as explanatory videos or expanded versions of booklet information, enhances the likelihood of messages being effectively communicated.

The prototype’s potential to present health information in different languages was highly appreciated by participants. They emphasised its importance for individuals whose first language is not English, allowing health information to be communicated in their native languages. This feature was considered valuable, especially as materials are not typically produced in multiple languages. Participants highlighted that the prototype could cater to a diverse range of preferences, technological engagements, languages and background, making health information more accessible to a broader audience.

A notable concern raised was the requirement for users to download an app to utilise the prototype. Participants expressed dislike for this aspect, as it could take up significant storage space on users’ devices. They suggested that engagement would likely improve if accessing the content did not depend on downloading an app, making the process more convenient and accessible.

**Best prototype aspects**

When discussing the best aspects of the demonstrated prototype, participants highlighted the advantages of augmented paper as a concept. They appreciated how it encourages users to access additional content they might not otherwise explore. The inclusion of videos and audio guides for exercises or activities, such as breathing exercises, was seen as particularly beneficial. They noted that auditory or visual instructions can often be clearer than written text, catering to those who learn better visually or audibly. At the same time, the printed pages serve as valuable reference material, providing a complementary resource for users.

The availability of both paper and digital mediums, without reliance on digital access, was seen as a key advantage. Participants appreciated that the printed health information could still benefit individuals who lack access to digital devices or the internet. Another highly valued feature was the ability to provide content in multiple languages, with participants noting the ease of accessing pages in any language. They also appreciated the option to have pages read aloud, and the app’s session-based structure was described as easy to navigate.

**Worst prototype aspects**

During the interviews, participants were asked about the negative aspects of the demonstrated prototype, and the primary concern raised by most was its lack of cost-effectiveness. They highlighted that producing two types of media to deliver information, printed and digital, would incur significant costs. Additionally, they emphasised that developing an app, along with the ongoing expenses of updates and maintenance could make it too expensive.

Participants also mentioned that requiring users to download an app might discourage them from accessing additional information. They expressed concerns that this extra step could be unnecessary, and that people may hesitate to download an app due to storage limitations on their devices. Instead, they suggested that similar access to information could maybe be achieved through alternatives like QR codes. They believed that health campaigns might see higher engagement if they did not depend on users having to download an app.

The lack of accessibility of this approach was identified as a significant obstacle. Participants noted that, while a significant portion of the population is digitally engaged, it cannot be assumed that everyone has access to a smartphone or digital device for health information. This limitation makes digital information inaccessible to some. Another drawback highlighted during the demonstration was the booklet used in the prototype, which occasionally featured multiple URLs on the same page. Participants felt this could be confusing and suggested that consolidating the web links into a single, streamlined link would be a more user-friendly alternative.

**Usability of the link between paper and screen**

When asked about the usability of the augmented paper approach, participants highlighted the benefits of linking paper and digital mediums. They appreciated the flexibility of having a physical copy of the information that can be taken anywhere without necessarily relying on digital content. At the same time, they valued the ability to access additional reliable digital information easily when desired. Participants noted that this method of directing people to supplementary content online is much quicker and more convenient than searching for it via search engines or manually typing URLs. This approach was seen as particularly effective in encouraging engagement with supplementary material without overwhelming readers with excessive information in a leaflet. Additionally, it was highlighted as especially useful for individuals who may find extensive reading challenging.

Participants noted that this approach could be particularly valuable for SCC health partners who produce a significant volume of printed materials. They also highlighted its potential usefulness for staff, such as social workers, midwives, and other health professionals, to provide more detailed explanations to the public when distributing leaflets. On a content level, participants suggested that this method would be especially beneficial for guiding physical exercises or activities in green spaces, such as countryside walks.

The following quote highlights a participant’s perspective on the potential benefits of integrating physical materials with digital technology to enhance health promotion efforts: *“The infrastructure and the concepts there, there’s no reason why it can’t be there for more like health promotion things. You know, you think you see something about if you’re a smoker, and you’ve seen a leaflet which is really enticing because the messaging is so strong. But actually, you don’t then want to bombard that leaflet with, ‘This is how you refer,’ and ‘This is your eligibility criteria.’ But then if there’s a QR code, it takes you straight there, and you go, ‘OK, yep, I’m eligible. Sign me up here, referral done.’ So I think you can then use that space on a physical something for more appropriate messaging. It goes back to that behaviour change, it’s going to kind of pull people in.”*

**Suggestions on how to improve the prototype**

A key suggestion for improving the demonstrated prototype was to maintain the concept of linking printed information with digital content while eliminating the need to download an app. Participants proposed integrating a technology like QR codes to streamline access. However, they highlighted the importance of organising the digital content effectively, given that each page contained multiple hot links. They suggested limiting each printed page to a single QR code, which would direct users to a list of content options on their digital devices for easier navigation. Another suggestion for accessing hotlinks between mediums was to incorporate this approach into an app that users are likely to already have on their phones, such as the NHS app

Participants suggested several content-level improvements for the prototype, emphasising the need for more user-friendly and engaging materials. They recommended incorporating specific infographics and providing audio descriptions for images and graphs to enhance accessibility for visually impaired users. Additionally, they proposed adapting this approach for smaller printed materials, such as leaflets, rather than focusing solely on larger booklets. The following quote illustrates how stakeholders prepare health information for audio transcription before making it available on their website: *“If we’re including a graph, then we’d have to say exactly what that graph is saying. Some graphs can be quite simple, but others that are more complex, like those with multiple lines, require accessing the original data to ensure it is fully explained and included within the copy as well.”*

**Other devices to display content**

When discussing alternative devices for displaying content with this approach, participants suggested large digital screens or TVs for use in health practitioner waiting rooms. Tablets were also mentioned, with their larger screens noted as an advantage for viewing content. However, participants observed that scanning on tablets might not be as convenient. Additionally, they emphasised the importance of ensuring compatibility with screen readers to enhance accessibility for users with visual impairments.

### 3.3 Results integration

This section integrates findings from the previous studies: focus groups with digitally engaged older adults and online interviews with health content producers from the stakeholder institution, to provide a nuanced understanding of participants’ attitudes towards health information strategies and delivery methods.

**Accessing and delivering health information**.

Both groups of participants consistently emphasised the growing importance of digital platforms for health information delivery, particularly highlighting the convenience of smartphones for quick access. In the focus groups, older adults identified mobile devices as their primary tool for quick health searches, particularly because of their constant availability. However, participants also mentioned using desktop or laptop computers for more thorough and serious searches, as the larger screens made it easier to process and read detailed information. Health content producers echoed the significance of mobile-first strategies, with one interviewee noting the high percentage of users accessing their content via mobile devices.

While the focus group findings highlight the tools older adults use to access health information, they also reveal that these individuals tend to search for health information only when they or someone they know is experiencing symptoms of a health issue. This contrasts with insights from the interviews with health content producers, who emphasised the importance of tailoring health information to meet the needs of different demographics. Producers highlighted how identifying these needs and developing effective strategies for delivering preventive health information are crucial for reaching this population before health issues arise. These findings underscore the importance of stakeholders proactively engaging older adults with preventive information to promote health and well-being, thereby reducing the likelihood of health issues developing.

**The evolving role of printed materials in health information access**.

Both studies highlighted a decline in the use of printed materials for health information. Focus group participants noted that while they don’t feel the need to receive all health information in printed form, they value having the option to choose which types of information they would prefer in print. Similarly, health content producers interviewed explained that in recent years, there has been a shift toward delivering health information primarily online due to its cost-effectiveness and broader reach. However, they continue to produce some printed materials to ensure accessibility for individuals who may lack internet access, digital devices, or remain digitally disengaged. Additionally, all their online content is made available in printable formats.

Despite the shift toward digital delivery, both studies emphasised the importance of maintaining non-digital options for sharing and accessing health information. Older adults in the focus groups expressed a preference for printed materials when it comes to important documents they want to keep, remember, or refer back to later. They also noted the ease of locating physical documents at home compared to searching for digital files. Similarly, health content producers acknowledged the need to offer multiple formats to reach diverse audiences, recognising that not everyone prefers or has reliable access to digital content.

**Value of the augmented paper approach**.

Both studies highlighted the importance of providing opportunities to access and share supplementary health information through a combination of paper and digital media. Focus group participants emphasised the benefits of using diverse media formats to cater to different learning preferences and offer varying levels of detail on health-related topics. They suggested that this approach could be particularly useful for educational purposes in healthcare settings, such as general practice (GP) clinics, or for understanding pension schemes offered by pension companies.

Similarly, health content producers interviewed viewed this approach positively, recognising the potential of delivering content through multiple media formats and at different levels of depth. They noted that this strategy could enhance audience engagement and improve the effectiveness of information dissemination by reaching a broader and more diverse public.

**App-less approach**.

Participants in both studies expressed a clear preference for an approach that eliminates the need to download an additional app. Across both methods, participants suggested using alternatives like QR codes or other simple mechanisms that would allow them to easily access digital content through printed materials without requiring an app on their devices.

**Ensuring user-friendliness and accessibility in health information**.

In both studies, participants stressed the importance of presenting health information in a user-friendly and accessible format. Focus group participants emphasised the need for content that is simple, intuitive, and engaging, making it easier for users to understand and retain information. Similarly, health content producers highlighted the importance of tailoring content to meet the needs of diverse audiences by providing it in multiple formats, such as digital and print, as well as visual and audio. They also suggested that all digital content should include the option of audio transcripts to enhance accessibility for individuals with visual impairments or those who prefer auditory learning.

**Preferred devices and accessibility considerations for health information delivery**.

When discussing the potential devices for displaying health content, participants in both studies suggested that TVs could be effective in group settings, such as educational sessions or GP waiting rooms. Focus group participants highlighted the benefits of larger screens, like those on laptops and desktops, for better visual clarity and ease of reading. They also expressed a preference for having the option to self-print relevant content they wanted to reference or consult later. In the interview study, content producers noted that most users accessed health information via smartphones and tablets. While tablets offer the advantage of larger screens, they acknowledged that scanning augmented content on these devices might be less convenient. They also emphasised the importance of designing content in accessible formats, ensuring compatibility with screen readers to support users with visual impairments.

### 3.4 User requirements for optimal health information delivery

The following table (Table 6) summarises the key user requirements identified across both studies, combining insights from the focus groups and online interviews. By synthesising feedback from older adults and health content producers, the table provides a comprehensive overview of the essential features and considerations for creating accessible, engaging, and user-friendly health content across different platforms. The table highlights the shared priorities and distinct considerations that emerged in both studies, offering valuable guidance for the development of effective health communication strategies. Following the table, each user requirement is explained to draw out their implications for health information design and dissemination.

**Table 6:** User requirements table.

User Requirement	Focus Group Findings	Interview Findings
**Optimised Use of Paper and Digital Formats**	Preference for reduced paper usage, with digital formats as the primary mode of access; option to print selected materials for reference.	Shift towards digital dissemination due to accessibility and cost-effectiveness; some printed materials remain necessary for individuals without internet access.
**Avoid Sole Reliance on Digital Formats**	Emphasis on maintaining non-digital options to ensure accessibility and tangibility.	Preference for a hybrid approach that includes supplementary digital content alongside printed materials.
**App-Free Access**	Preference for QR codes or scanning methods to access digital content without requiring app downloads.	QR codes as a bridge between physical and digital content; download of an app considered unnecessary step.
**Integration with Smaller Printed Materials**	Augmentation preferred for smaller printed documents, such as leaflets, rather than extensive booklets.	Augmented materials suggested for health promotion, including physiotherapy exercises and NHS leaflets.
**Accessibility Considerations**	Need for user-friendly, intuitive formats.	Emphasis on accessibility, including user-friendly design, audio transcriptions, and screen-reader compatibility.
**Device Compatibility**	Preference for screen casting to televisions for group settings; access via laptops and desktops for in-depth searches and careful reading.	Television screens recommended for group settings and educational purposes.
**Additional Content in Multiple Formats**	Valued supplementary content in various formats (e.g., videos, images, audios) to accommodate different learning styles and provide varying levels of depth.	Multi-format content (e.g., videos, infographics) seen as beneficial for engagement and comprehension.
**Option for Self-Printing**	Printed materials preferred for careful reading and long-term reference; printing options necessary for key documents.	Digital materials should be available for self-printing.
**Trust and Credibility**	Both digital and printed content should originate from a credible source and feature clear branding to enhance trustworthiness.	Not Applicable.
**Cost-Effectiveness**	Not Applicable.	Digital formats are preferred for their cost efficiency and ease of dissemination; however, printed materials remain essential. A cost-effective approach should integrate both formats seamlessly.

- **Optimising the Use of Paper and Digital Formats:** Both studies emphasised a preference for reducing reliance on printed materials while maintaining the option to print specific information. This requirement highlights the importance of integrating digital and print in a way that maintains accessibility without increasing costs.
- **Avoiding Over-Reliance on Digital Platforms:** Participants in both studies stressed the need to provide health information through multiple channels rather than relying exclusively on digital formats. This finding underscores the necessity of a hybrid approach to ensure equitable access to health information.
- **App-Free Accessibility:** A recurring theme was the preference for accessing digital health content without the need to download an additional app. This aligns with broader concerns about digital literacy and the potential barriers posed by requiring users to navigate unfamiliar applications.
- **Augmented Leaflets and Smaller Printed Documents:** Participants across both studies suggested that smaller printed materials, such as leaflets, should be augmented with digital content rather than larger booklets.
- **Enhancing Accessibility and User-Friendliness:** Both focus groups and interviews underscored the importance of making health information user-friendly and accessible. Audio transcriptions and screen-reader compatibility were particularly noted as crucial for ensuring accessibility among visually impaired users and those who prefer auditory learning.
- **Flexible Device Compatibility:** Participants emphasised the need for flexibility in accessing digital content, highlighting the importance of ensuring compatibility across various devices. They noted that larger screens, such as those on laptops, desktops, or TVs via screen casting, can enhance readability and be more feasible for group settings.
- **Additional Content in Multiple Formats:** Participants highlighted the value of providing supplementary information in various formats to accommodate different learning preferences
- **Self-Printing Options:** The ability to print materials on demand emerged as a key requirement in both studies. This reinforces the importance of a flexible approach that allows users to choose their preferred format based on their needs.
- **Building Trust Through Reliable Sources:** Focus group participants stressed the importance of clear branding and credibility to reinforce confidence in digital and printed materials
- **Cost-Effectiveness and Sustainable Integration:** Content producers highlighted that digital formats are more economical and easier to disseminate. However, both studies acknowledged that printed materials remain necessary, particularly for those without digital access. The challenge lies in finding a sustainable, cost-effective method to integrate both formats while ensuring that information remains accessible and up to date.

## 4. Discussion

This study explored the complexities of health information consumption and production to older adults in an increasingly digital context. In relation to RQ1 (older adults’ preferences for accessing health information), our findings confirm the migration from paper to digital formats, while also showing that printed materials remain valued for their familiarity, tangibility, and perceived reliability. With regard to RQ2 (content producers’ perspectives), professionals acknowledged similar challenges and highlighted the need for adaptable communication strategies that address diverse public demands. Finally, RQ3 (the role of augmented paper as a hybrid solution) was addressed through the identification of key user requirements for bridging paper and digital formats, including flexible access to content, usability considerations, and trust-building mechanisms. Together, these insights suggest that augmented paper represents a promising approach to integrate the advantages of both mediums, offering a hybrid solution that aligns with the evolving needs of older adults while maintaining accessibility and engagement.

Concerning the current consumption of health education, a key finding from this study was that older adults primarily seek health information online in a reactive manner, only when they experience symptoms or specific health concerns, a finding that aligns with Zhao et al. (2022) [14], who found that older adults often search for health information to gain a general understanding of medical conditions before seeking professional diagnosis or treatment, but not preventively. Additionally, this trend reflects the broader transition from paper-based to digital health information, as highlighted in the studies by Ferraz et al. (2024) [4] and Aslan et al. (2024) [5]. While digital platforms offer greater accessibility and a wider range of information, a major concern raised by participants in this study was the credibility and trustworthiness of online health content, as they reported difficulties in distinguishing reliable sources from misinformation. Similarly, Hu et al. (2024) [15] highlighted the widespread prevalence of health misinformation among older adults across countries with varying levels of development. These findings underscore the urgent need for stakeholders to promote and encourage preventive health information for older adults, enabling them to make informed decisions about their lifestyle before health issues arise. However, these efforts must also address the serious challenge of misinformation, ensuring that older adults have access to accurate, reliable, and easily comprehensible health content.

Regarding the production of health content, a key finding from our study with content producers at SCC was that they are also transitioning to digital formats for delivering health information. However, accessibility is a major concern, and they still emphasise the need to provide printed content for those unable to access digital formats. The literature highlights that technology-based interventions are particularly effective in improving health literacy [16], as they offer easier access, better engagement, and more personalised content. Integrating digital tools with simple printed information could increase engagement, while ensuring accessibility for those without digital access.

Both studies in this paper support the feasibility of combining paper and digital formats for health education. A scoping review on older adults’ preferences for patient health materials found that they favour hard copy handouts or downloadable formats [17]. The same review highlights that older adults primarily obtain patient education materials from healthcare professionals or the internet. However, while many turn to online sources for additional information, they often struggle to identify reliable resources and largely expect healthcare providers to guide them toward credible information [17,18]. Additionally, concerns about the accuracy and reliability of online health content contribute to hesitation and low trust in digital health information. These findings reinforce the value of printed materials for their tangibility and perceived trustworthiness while also recognising the benefits of digital content for supplementary information. Augmented paper presents a promising solution by integrating both formats, allowing healthcare professionals to provide printed materials that link to carefully curated, reliable online resources. This approach ensures that older adults have access to trustworthy, accessible health information while accommodating their diverse preferences and digital literacy levels.

### Possible design trajectories

Both studies in this paper conducted with potential users and stakeholders, highlighted the feasibility of using augmented paper with QR codes for health information, particularly through an app-free approach. QR codes have shown significant potential in healthcare, improving patient engagement, streamlining workflows, and enhancing accessibility to medical information. A recent comprehensive review on the use of QR code for healthcare [19] underscores their efficiency in providing quick access to medical resources, ensuring medication safety, and supporting patient education. Additionally, QR codes offer security and privacy advantages by enabling controlled access to medical records. However, challenges remain, including implementation delays due to certification requirements, technical issues such as image distortion, and security vulnerabilities. Adoption barriers also persist, as both healthcare professionals and patients may be reluctant to use QR codes for data access and authentication.

Despite these challenges, QR codes present a promising solution for bridging paper and digital formats in health communication. However, unlike mobile applications, QR codes typically direct users to a single URL, which may limit access to multiple media formats. The prototype developed in this study demonstrated the potential for providing multiple media options. Future research could explore tools such as Linktree, a content aggregation platform that consolidates multiple web links into a single, easily accessible landing page, or similar platforms to optimise the delivery of health information through QR code. Such tools could enhance the accessibility and organisation of digital resources by allowing one printed QR code to provide structured access to a range of materials.

While QR codes and options for self-printing were widely supported by participants, these approaches may still present barriers for older adults with minimal digital literacy or no access to smartphones or printers. For such groups, reliance on QR codes could inadvertently reinforce exclusion rather than overcome it. Future implementations of augmented paper should therefore be accompanied by alternative, non-digital access points to ensure inclusivity across the spectrum of digital confidence and device availability.

In addition, we recognise that our sample was relatively homogeneous and skewed toward older adults who were already moderately engaged with digital technologies. This introduces selection bias that may limit the transferability of our findings. Our recommendations should thus be interpreted as reflecting the preferences of digitally engaged older adults in a UK setting, rather than the full diversity of older populations. Broader testing across varied socioeconomic and cultural contexts will be essential to refine these strategies and ensure their wider generalisability.

The transition from paper to digital health records is widely seen by hospitals and clinical practices as an inevitable progression, with the expectation that health records will eventually become entirely digital [20–22]. However, our study suggests that older adults do not fully embrace this shift. Rather than viewing digital formats as a complete replacement, they preferred to access health information online first and then print only what they consider essential. They were comfortable receiving less printed material, provided they could access comprehensive and supplementary content digitally. This preference presents a cost-effective opportunity for stakeholders, as minimal printed materials can serve as a structured index, linking to detailed, updatable online resources. Future research should explore how augmented paper techniques can further optimise this hybrid approach, balancing digital accessibility with the practical advantages of printed formats.

Beyond this selective use of printed materials, our findings also highlight the appeal of self-printing and custom-printing options. Rather than relying on pre-printed health documents, older adults valued the ability to curate and print only the information most relevant to them. This reflects a shift in the role of printed materials from a universal, static resource to a personalised, supplementary tool. Previous research confirms that older adults continue to favour tangible health education materials while also benefiting from digital accessibility [17]. Incorporating self-printing with augmented paper strategies could enhance engagement while reducing printing and distribution costs for healthcare providers. Future research should explore how healthcare providers can support this hybrid approach, offering curated, printable resources alongside reliable digital content.

A key limitation of this study is the recruitment criteria, which included only older adults with smartphones or tablets. As a result, the findings may not fully represent the experiences and preferences of those who are less digitally engaged or who do not use smartphones at all. Additionally, the recruitment process likely favoured participants who were already comfortable with digital platforms, as the recruitment form was distributed online. This may have introduced a selection bias, meaning that perspectives from individuals who primarily rely on printed health information or have limited digital literacy were underrepresented. If anything, augmented paper may be considered to be even more valuable to this group as a kind of on-ramp to curated digital information, perhaps viewed on communal devices or home TVs. Future research should explore the perspectives of digitally excluded older people and alternative implementations of augmented paper beyond smartphones.

The user requirements identified through the thematic analysis of both studies highlight the opportunity of a hybrid approach to health information delivery. Integrating digital and print resources in a way that is accessible, engaging, and cost-effective can better accommodate diverse user preferences while enhancing the reliability and usability of health information. This approach ensures that while digitally engaged individuals can benefit from interactive and updatable online content, those who are unable to access digital resources will still receive essential printed materials. By adopting this balanced strategy, stakeholders can make health communication more inclusive, ensuring that technological advancements do not exclude individuals who rely on tangible formats. Health information should remain accessible to all, regardless of their level of digital engagement.

## Supporting information

S1 Appendix Prototype description and technical features. (DOCX)
