# Supplementary material for: Optimising the provision of health information for older adults across paper and screen formats – A requirement study with content producers and consumers
Source: PLOS Digit Health. 2025 Nov 17;4(11):e0001090. doi: 10.1371/journal.pdig.0001090 (PMC12622786; doi:10.1371/journal.pdig.0001090)
Supplement: S1 Appendix — (DOCX) [file pdig.0001090.s001.docx]

**S1 Appendix. Prototype Description and Technical Features**

**Prototype Overview**

The study used the **Next Generation Paper (NGP) platform**, which enables the augmentation of printed materials with digital content. A printed health information booklet titled *First Steps: Emotional Health and Mental Well-Being*, produced by Surrey County Council (SCC) in collaboration with the National Health Service (NHS), was selected as the source material. This booklet was suitable for augmentation because it contains preventive health information and multiple embedded web links.

**Authoring Application**

The **NGP Authoring App** is a private, Android-only application that allows researchers and practitioners to augment existing pages of a printed booklet with multimedia content. Multimedia options include images, audio, video, and hyperlinks. The app was used to embed supplementary resources into the *First Steps* booklet for the purposes of the prototype.

**Player Application**

The augmented paper **NGP Player App** is cross-platform, available on both Google Play (Android) and the Apple App Store (iOS). It provides multiple navigation modes for accessing linked digital content from an augmented booklet:

- **Visual recognition**: capturing an image of the printed page with the app’ camera
- **Voice recognition**: speaking the page number to the app.
- **Manual entry**: typing the page number directly using the app interface.

Once a page is recognised using one of the above modes, the app displays a list of linked digital items, which can be opened on the user’s device. Additional features include bookmarking pages for future reference.

**Demonstration Materials**

For both focus groups and interviews, the augmented booklet was introduced using the same project demonstration. This ensured consistency across data collection sessions.

- **Focus groups**: researchers presented the prototype with a scripted live demonstration, followed by individual participant exploration.
- **Interviews**: participants were shown a demonstrative video of the prototype before providing feedback.
